# The Effect of Selenium–Arabinogalactan Nanocomposite on Fatty Acid Composition in Soybean Seedlings Grown from *Pectobacterium carotovorum*–Infected Seeds

**DOI:** 10.3390/plants15111647

**Published:** 2026-05-27

**Authors:** Alla I. Perfileva, Natalia V. Semenova, Elena Yu. Garnik, Alla V. Korobova, Nadezhda V. Klushina, Boris G. Sukhov, Irina S. Kapustina, Vadim N. Nurminsky

**Affiliations:** 1 Siberian Institute of Plant Physiology and Biochemistry, Siberian Branch of Russian Academy of Sciences, 664033 Irkutsk, Russia; alla.light@mail.ru (A.I.P.); tashasemyonova@mail.ru (N.V.S.); elga74@yandex.ru (E.Yu.G.); nirinka24@mail.ru (I.S.K.); 2 Institute of Biological Sciences, Irkutsk State University, 664003 Irkutsk, Russia; 3 Ufa Institute of Biology, Ufa Federal Research Centre, Russian Academy of Sciences, 450054 Ufa, Russia; muksin@mail.ru; 4 Laboratory of Nanoparticles, V.V. Voevodsky Institute of Chemical Kinetics and Combustion, Siberian Branch of Russian Academy of Sciences, 630090 Novosibirsk, Russia; klushinanadezda@kinetics.nsc.ru (N.V.K.); boris_sukhov@mail.ru (B.G.S.)

**Keywords:** abscisic acid, gene expression, *Glycine max*, desaturases, nanoparticles, saturated and unsaturated fatty acids, phytopathogenic bacteria, priming, seeds

## Abstract

The phytopathogenic bacterium *Pectobacterium carotovorum* (*Pcc*) infects a wide range of crop plants and causes substantial economic losses. The authors of this study previously demonstrated that the selenium–arabinogalactan nanocomposite (Se/AG NC) is capable of mitigating the negative effects of infection of soybean seeds with *Pcc* during germination and can influence physiological and biochemical factors in the seedlings. This study investigated changes in the membrane fatty acid (FA) profile of soybean seedlings grown under different treatments and in control using chromatography–mass spectrometry (CMS). The soybean seed treatments included the following: (1) infection by *Pcc* alone; (2) nanopriming with Se/AG NC alone; and (3) infection by *Pcc* followed by nanopriming with Se/AG NC. The infection was performed by soaking seeds in a bacterial suspension. Nanopriming was performed by placing the seeds in an aqueous solution of Se/AG NC (6.25 µg/mL) with a Se concentration of 0.000625%. Then, the seeds were germinated over 5 days in the darkness at 25 °C. The FA profile of soybean seeds was characterized by 13 FAs dominated by linoleic (LA), linolenic (LNA), oleic (OA), palmitic (PA) and stearic (SA) acids. Se/AG NC nanopriming had no influence on the FA profile of soybean seeds. A unique FA profile of soybean seedlings was demonstrated. It consisted of 18 FAs containing 12 to 20 carbon atoms. The following FAs were dominant in the control samples: PA (28%), LA (32.8%), LNA (18.6%), and SA (7.5%). *Pcc* infection of the seeds amplified the content of unsaturated FAs. Nanopriming of the seeds with Se/AG NC had an obvious influence on the seedling FA profile. Treatment of soybean seeds infected with *Pcc* using Se/AG NC caused weakening of the detrimental effects of the pathogen, while giving the possibility to maintain soybean seedlings’ FA profile at the control level. Transcript levels of the *GmFAD8-2* gene encoding the membrane-bound omega-3 FA desaturase (FAD) were elevated for soybean seedlings after both *Pcc* and Se/AG NC seed treatment processes. The FA double-bond index (DBI) grew under the influence of seed infection and dropped under other treatments. Nanopriming of the seeds with Se/AG NC effectively reduced stress in *Pcc*-infected plants, as evidenced by analysis of the abscisic acid (ABA) content. Variations in the membrane FA composition under nanopriming with Se/AG NC may be one of the forms of its phytoprotective effect.

## 1. Introduction

Diseases of crops, which manifest as rotting, are still widespread despite the advances associated with the usage of various disease-resistant varieties and fungicides. Gram-negative motile bacterium *Pcc* is one of the rot-causing phytopathogens [1]. During post-harvest processing, many cultivated plants (including such vegetables as Chinese cabbage, lettuce, radish, potato, tomato, onion, etc.) are susceptible to soft rot as a result of this pathogen’s activity [2]. *Pcc* secretes a wide range of enzymes, which break down plant cell walls, including pectinases, polygalacturonase, cellulases, and proteases that cause softening and disintegration of plant tissues [3]. A negative influence of *Pcc* on soybean seedling viability (as earlier reported in [4]) has also been verified in our investigations [5]. This might have been a problem because soybeans play a vital role in global food safety. Soybeans are among the five most widely cultivated crops in the world [6]. At the same time, there is a burgeoning research drive to discover and implement new, environmentally friendly agents that can improve plant survival against *Pcc*. The pesticides currently in use are designed to manage populations of disease-causing fungi, but they lack efficacy against bacterial infections.

Seed priming may be considered (in some sense) as an alternative to the application of pesticides. But traditional methods of pre-sowing seed treatment have a number of limitations, which reduce their efficiency and practical applicability [7]. These involve various kinds of seed treatment, which initiate the germination before planting, such as hydropriming, osmopriming, thermopriming, biopriming, and chemopriming [8,9]. Seed pre-treatment using nanoparticles (NPs) (nanopriming) gives promising perspectives, while demonstrating positive effects expressed in the growth of the yields and enhancement in resistance of the plants to stress factors [10,11,12,13,14]. A key benefit of nanopriming consists in its potential association with a reduction in the burden of reactive oxygen species (ROS) in seeds experiencing stress during germination [13,14,15]. As a result, the formation of lipid peroxidation (LPO) products decreases, which is considered an important aspect of seed health [16]. Earlier, in our experiments, we discovered that soybean seedlings subjected to nanopriming had better biometric characteristics compared to the controls. Equally, the negative effect of *Pcc* on soybean seedlings decreased under the influence of NC, while retained was some effect on the activity of antioxidant enzymes and on the content of LPO products. FAs are the fundamental elements of phospholipids, which represent the “building blocks” of cell membranes, and, normally, FAs fulfill a structural function. Meanwhile, this is not their only role in the plant cell. In this connection, it is crucial to determine the impact of NC on the FA profile of soybean seedlings subjected to *Pcc* infection.

The level of diene conjugates (DCs) was lowered under the conditions of biotic stress [5]. DCs are the compounds formed as a result of oxidation of polyunsaturated FAs (PUFAs) [17,18]. PUFAs are subjected to oxidation, which can take place both as a result of enzymatic reactions catalyzed by lipoxygenases and as a result of non-enzymatic reactions initiated by ROS [19,20]. FA oxidation triggers the synthesis of numerous oxylipins, which have various biological roles in plants [21]. Some of these oxylipins are characterized by direct antimicrobial properties, while others act as potent regulators of defense mechanisms [22]. Furthermore, oxylipins represent some part of the complex inter-organismal phytohormone networks, which include such important components as salicylic acid, ethylene, auxin, brassinosteroids, gibberellic acid, and ABA. The phytohormones control all the factors influencing the growth and development of plants, along with their capacity to adjust to environmental changes [23]. Therefore, it is of interest to evaluate how the ABA content varies during nanopriming under the conditions of biotic stress, which is a growing trend in contemporary directions of research.

Under the influence of various factors, the FA composition of membrane lipids in shoots and roots of plant seedlings changes. The ratio of saturated (SFAs) and unsaturated (USFAs) membrane FAs is regulated by special desaturases, enzymes changing the degree of FA saturation [24]. Investigation of the FA composition allows us to assess the indicator of fluidity of cell membranes. In turn, fluidity determines the state of membrane proteins. Correct functioning of these proteins is considered to be quite important for normal seed germination and subsequent development of the plant [25]. Investigations concerning FAs and their metabolic pathways are crucial in numerous areas of biology. Specific FAs and their proportions in cell membranes can serve as biomarkers for the identification of organisms and for the purpose of investigation of the bacterial cell adaptation to toxic compounds and heavy environmental conditions [26].

Furthermore, soybeans represent the source of a valuable food product—soybean oil. Therefore, improving the FA composition is an important aspect of growing this crop [27]. Soybean oil consists of five main FAs, i.e., LA (C18:2), OA (C18:1), PA (C16:0), LNA (C18:3) and SA (C18:0), approximately in proportions of the total FA content equal to 55%, 18%, 10%, 13% and 4%, respectively [28]. To improve the quality of soybeans, various genetic techniques were developed and used [28,29,30]. In this connection, it is of interest to determine how nanopriming of soybean seeds with Se/AG NC influences the plant’s FA composition, both in seeds and in seedlings. Noteworthily, most of the published results of investigations dealing with the FA profile of soybeans focus primarily on the analysis of the five main FAs and do not represent the full spectrum of FAs.

So, any pithy investigations addressing metabolic processes in connection with FAs, their oxidation and their influence on plant cells may be considered as opening new frontiers in comprehending the processes by which plants adapt to challenging conditions and development of new techniques for elevating their resilience for improving crop productivity in the face of climate changes and emerging food shortages. Therefore, the purpose of the current study was to analyze the variations in the FA composition of lipids from soybean seedlings (i) under the condition of infection with the phytopathogenic bacterium *Pcc* and (ii) treated with Se/AG NC. Furthermore, the expression of several genes encoding desaturases was assessed.

## 2. Results

### 2.1. Characteristics of NC

**Figure 1:**
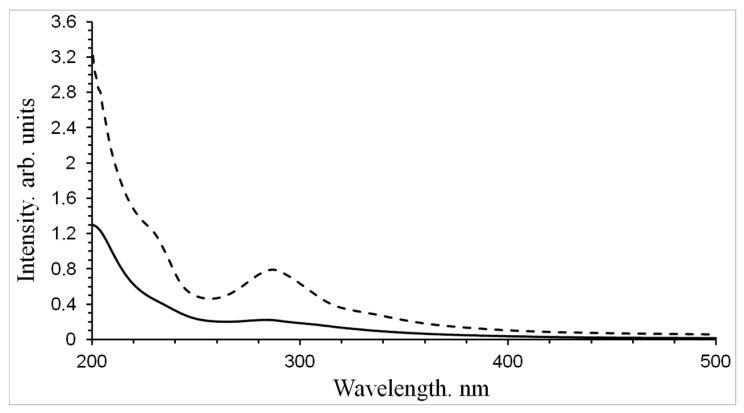
Optical absorption spectra of the original raw material of AG polymer (dashed line) and Se/AG NC (solid line).

It was previously shown in investigations that natural macromolecules of arabinogalactan (AG) of Siberian larch represent molecular clathrates, i.e., inclusion compounds of the “host-guest” type, with the inclusion of about 5% by weight of bioflavonoids in AG macromolecules, the dominant of which is dihydroquercetin [31]. In the present investigation, we intended to use these flavonoids as a reducing agent in the reaction with selenious acid and the resulting formation of NPs of elemental selenium (Se) directly in AG macromolecules [32]. In order to obtain Se NPs, a sample of selenious acid was taken on account of the consideration that there was one Se atom per conventional molecule of flavonoid (dihydroquercetin, quercetin, etc.). Elemental analysis has shown the expected Se content in the NC is equal to 0.38%. For the Se/AG NC, a substantial decrease in the optical absorption maxima observed for the original preparation of AG was recorded (Figure 1).

**Figure 2:**
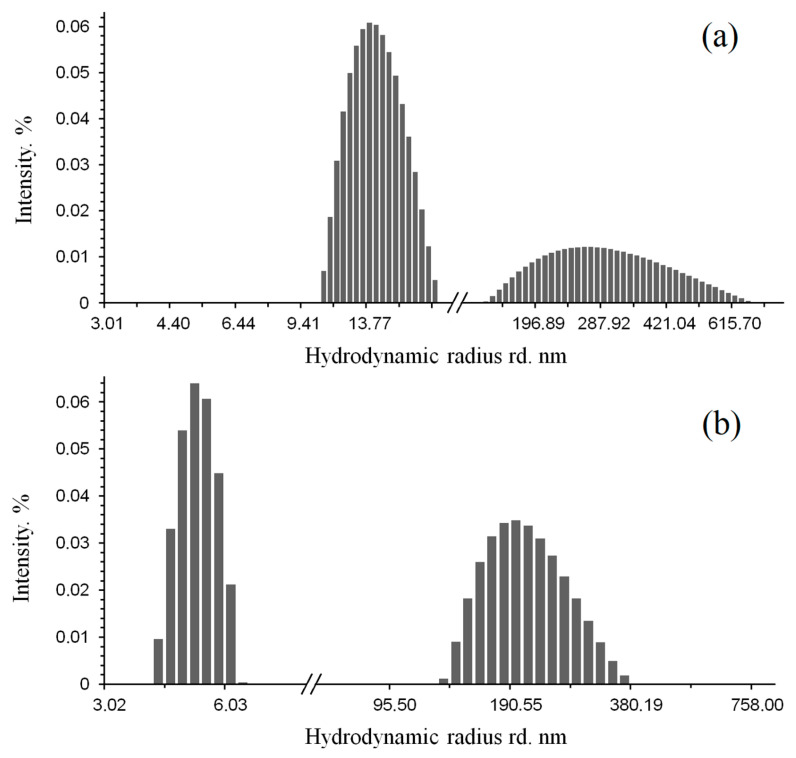
Distribution of hydrodynamic dimensions of the original raw material of AG polymer (**a**) and Se/AG NC based on the raw material of AG containing Se NPs (**b**).

The light scattering results show that in aqueous solution, the macromolecular size distribution of the original raw material of AG is bimodal, represented by fractions with average hydrodynamic radii of 14.7 nm and 312.3 nm (Figure 2a). This bimodality is also observed in the resulting NC. However, in this case, both fractions are reduced in the average radius: the small fraction, to 5.2 nm, and the large fraction, to 214.0 nm (Figure 2b).

### 2.2. Variations in the FA Composition of Soybean Seeds After Nanopriming

We hypothesize that Se/AG NC may influence the FA content in soybean seed tissues in a short time period after treatment.

**Figure 3:**
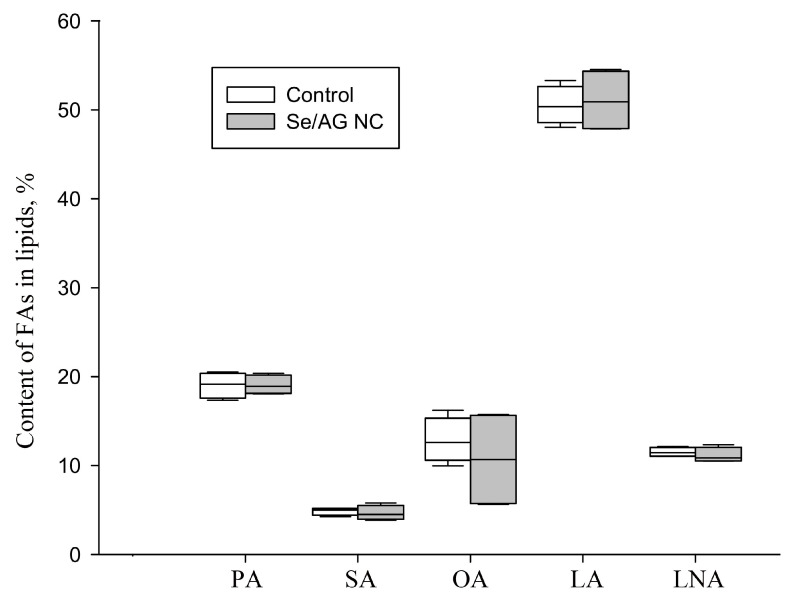
Content of essential FAs in the control soybean seeds and soybean seeds treated with Se/AG NC. LA—linoleic acid, LNA—linolenic acid, OA—oleic acid, PA—palmitic acid, SA—stearic acid.

This situation could have important practical implications for soybean oil quality modeling. Therefore, in the initial stage of our investigation, we determined the FA content of soybean seeds (i) for the control and (ii) after soaking in solution of Se/AG NC. The complete soybean seed FA content and the results of the effect of nanopriming on the FA profile of soybean seeds are shown in Table S1. Figure 3 shows changes in the content of the most important FA of soybean seeds after the nanopriming with Se/AG NC. In soybean seeds, it was LA that had the highest content while reaching 50%. LNA and PA were also high. Se/AG NC did not influence their content. However, under the influence of this NC, cis-vaccenic acid was revealed in the FA content, which was not observed in the control seeds (Table S1).

### 2.3. Variations in the FA Composition of Soybean Seedlings Under Biotic Stress and Nanopriming

In order to identify the mechanisms of influence of soybean seed infection with *Pcc* and nanopriming with Se/AG NC, variations in the lipid FAs of soybean seedlings have been investigated (Table 1). The results demonstrate that both control and treated soybean seedlings have the proper characteristics of qualitative and quantitative FA compositions.

The FA profile of soybean seedlings was represented by saturated branched-chain FAs (Ci12:0; Ca15:0; Ci15:0; Ca17:0), saturated straight-chain FAs (C14:0; C15:0; C16:0; C17:0; C18:0; C20:0; C22:0; C23:0), monoene FAs (C16:1 (n-7), 16:1 (n-9); C18:1 (n-7), 18:1 (n-9)), diene FA (C18:2 (n-6)), and triene FA (C18:3 (n-3)) (Table 1). Among them, in the control, C18:2 (n-6) (32.8%), C16:0 (28%), C18:3(n-3) (18.6%) and C18:0 (7.5%) dominated (Table 1).

Infection of soybean seeds with the phytopathogen *Pcc* contributed to the appearance of C20:1(n-11) and C17:0-i in the FA profile. Furthermore, this infection resulted in a substantial lowering of the content of saturated branched-chain FA C15:0-a, monodienoic acid (C16:1(n-9)) and saturated straight-chain FAs C17:0 and C22:0. At the same time, being compared to the control, some substantial and reliable growth in the content of diene FA C18:2(n-6), as well as an elevation in the content of saturated straight-chain FA C20:0, was noted. Variations in the total SFA and USFA were also pronounced. For example, ΣUSFA grew substantially. Furthermore, infection resulted in the increase of DBI and SDR in the tissues of soybean seedlings (Table 1).

We observed that FA C16:1(n-7) was missing under the conditions, when Se NC was present. Obvious growth in the contents of saturated branched-chain FA C15:0-i, monoene FA C18:1(n-9), and saturated straight-chain FAs C15:0, C20:0 and C22:0 was observed. Compared to the control, the contents of C16:1(n-9) and C18:3(n-3) were lower. However, an increase in SDR was observed in this treatment. DBI and ODR were lower than those in the control (Table 1).

It is obvious that nanopriming of *Pcc*-infected seeds neutralizes the effect of infection. Hence, lowering of the content of FAs C16:1(n-9) and C17:0 compared to the control with infection was less pronounced after nanopriming, and in some cases (FA C15:0-a, C17:0, ΣisoFA), their content was even higher than those in the control. The saturated branched-chain FA content of C15:0-i grew substantially in comparison to the control. This kind of treatment also demonstrated a decrease in the content of DBI and ODR, compared both to the control and to the treatment with *Pcc* infection.

### 2.4. Expression of Desaturase Genes in Soybean Seedling Tissues Under Biotic Stress and Nanopriming

**Figure 4:**
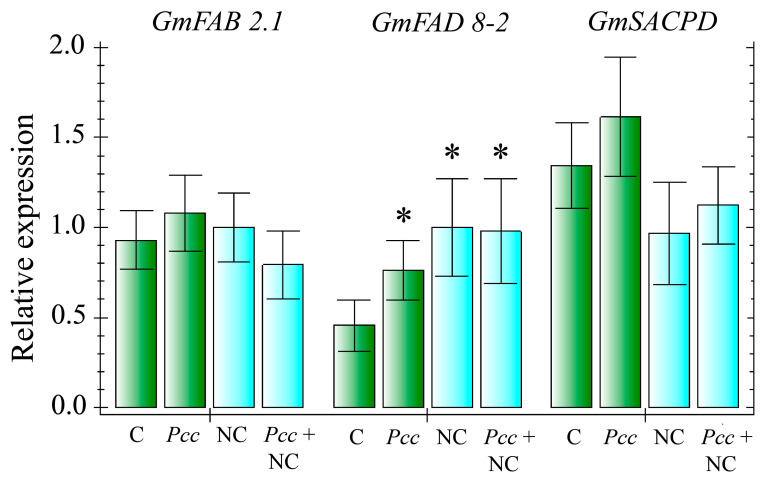
The transcript levels of the genes *GmFAB2.1*, *GmFAD8-2* and *GmSACPD* in the tissues of soybean seedlings sprouted from seeds infected with *Pcc* and treated with Se/AG NC. M ± S.D. (*n* = 5). *n* is the number of independent experiments in which each sample consisted of material from 15–25 seedlings. C—control; *Pcc*—seedlings grown from *Pcc*-infected seeds; NC—seedlings grown from the seeds nanoprimed with Se/AG NC; *Pcc* + NC—seedlings grown from *Pcc*-infected seeds and nanoprimed with Se/AG NC. * significant differences at *p* < 0.05 compared to control.

Most of the changes identified in the FA profile may be caused by desaturase activity, and substantial variations were observed in the dominant SFAs. Therefore, the expressions of the desaturase genes *GmFAB2.1*, *GmFAD8-2*, and *GmSACPD* were analyzed. The following results were obtained (Figure 4).

The transcripts of the *GmFAD8-2* gene grew twofold after Se/AG NC nanopriming, both in the absence and in the presence of *Pcc*. An elevated *GmFAD8-2* transcript level was also observed after *Pcc* infection without Se/AG NC (Figure 4).

### 2.5. The Content of ABA in Tissues of Soybean Seedlings Under Biotic Stress and Nanopriming

**Figure 5:**
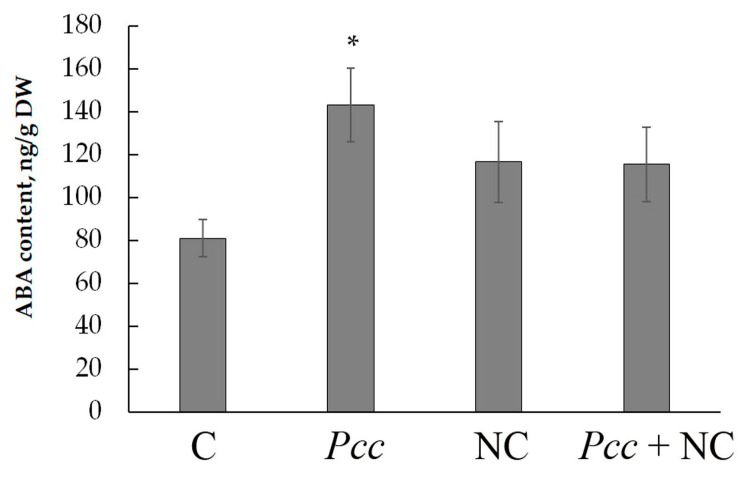
The ABA content in the tissues of soybean seedlings grown from *Pcc*-infected seeds and primed with Se/AG NC. M ± S.D. (*n* = 5). *n* is the number of independent experiments, in which each sample has been comprised by materials from 5–8 seedlings. * Statistically different averages (*p* < 0.05; ANOVA). C—control; *Pcc*—seedlings grown from *Pcc*-infected seeds; NC—seedlings grown from the seeds nanoprimed with Se/AG NC; *Pcc* + NC—seedlings grown from *Pcc*-infected seeds and nanoprimed with Se/AG NC.

Phytohormones play a crucial role in the regulation of various physiological and biochemical processes in plants under optimal and stress conditions [33]. ABA is a phytohormone involved in the regulation of vital physiological processes in the plant organism, one of which is the initiation of protective reactions against stress factors. Therefore, in the next stage of our investigation of the phytopathosystem, we analyzed the ABA content of soybean seedling tissues (Figure 5).

The results show that infection elevated the hormone level twofold compared to the control. Obviously, nanopriming of seeds with Se/AG NC was not stressful for the plant, as evidenced by the absence of elevation in ABA levels. In the treatment with *Pcc* infection and Se/AG NC, the ABA content did not increase substantially, which might indicate a reduction in the stress level in the plant. This was reduced compared to the treatment with *Pcc* infection alone (Figure 5).

## 3. Discussion

In the present investigation, a Se/AG NC with a Se NP content of 0.38% was synthesized, which is an easily reproducible result described in the patent [32]. In the process of comparing the optical absorption spectra of aqueous solutions of the original raw AG and the Se/AG NC obtained on this basis, which have similar concentrations, a clear difference in the intensity of the optical absorption bands of flavonoids in the ranges of 225–330 nm was observed [34]. This directly indicates the expected participation and consumption of flavonoids in the process of selenious acid reduction to elemental Se NPs. Obviously, the observed bimodal light scattering of NC may be explained by the participation of polysaccharide macromolecules competing with flavonoids as a reducing agent (these undergo oxidation) in the reaction of Se NP synthesis, with a loss of their mass and, consequently, the size of the macromolecules. Furthermore, in the case of the fraction with a small hydrodynamic radius, the remaining small fragments of macromolecules are likely no longer capable of holding and stabilizing nanoparticles of comparable size and remain free (without NPs) in the aqueous solution. Accordingly, only the second fraction of AG macromolecules with a larger hydrodynamic radius participates in the stabilization of the NPs.

### 3.1. Changes in the FA Composition of Soybean Seeds After Nanopriming

Se NPs are capable of stimulating the growth and development of soybeans. It was shown that Se NPs synthesized with the use of mycobacteria (in concentration of 1 μM) improve soybean seed germination, while elevating germination energy, germination rate and average germination time [35]. Transcriptomic analysis in soybean root and seedling tissues revealed six leading genes related to Se metabolism, such as 5′-adenylylsulfate reductase, methionine-tRNA ligase and chloroplastic NIFS-like cysteine desulfurases. The authors demonstrated that these genes play a key role in the accumulation of Se NPs and soybean resistance to them [36]. However, the physiological and biochemical mechanisms of the Se NP, which influence the soybean organism, were investigated insufficiently.

In the first stage of our investigation of Se NPs’ biological activity, their effect on the FA content in seeds was analyzed. In the control seeds, 12 FAs were identified. When treated with the NC, cis-vaccenic FA appeared in seed tissues. Other researchers also identified cis-vaccenic acid in plant seeds [37]. The authors suppose that the functional role of this FA is associated with a reduction in oxidative stress in cells. Cis-vaccenic FA has high pharmacological value due to the anti-inflammatory and antioxidant effects it produces. It also has a beneficial influence on the lipid metabolism in humans and animals [38]. So, the results show that nanopriming with Se/AG NC is capable of influencing the quantitative FA composition of soybean seeds. These results align well with earlier published information.

### 3.2. Changes in the FA Composition of Soybean Seedlings Under Biotic Stress and Nanopriming

This study is part of a series of investigations devoted to the biological effects of the NC on cultivated plants. In the initial stage, our investigations dealing with the influence of NC on plants were conducted on potato plants *in vitro*. Since the standard recipe of the Murashige-Skoog (MS) nutrient medium recommended for potato cultivation did not contain any source of Se, the Se/AG NC needed was introduced (added) into the MS medium of the standard composition (of the concentration of 0.000625%, previously selected experimentally), under which nanocomposites caused the desired antibacterial effect on phytopathogenic bacteria and did not influence the plant adversely (and, moreover, in some cases, even stimulated growth of the plants, their development and resistance to phytopathogens) [39]. After growing the plants on such a medium, an elemental analysis EDXMA of potato tissues was conducted. It was found that the Se content in leaf tissues of potato ranged from 0.01% to 0.03% of the air-dry mass. This observation pointed to both the penetration of Se into plant tissues from the NC and the subsequent, albeit minor, accumulation of Se within the plant after its treatment with Se-containing NC [40]. Another confirmation of the fact that nanoparticles are absorbed by the plant from the NC may be found in the results described in [41]. In that case, potato plants were grown on an MS medium, in which only NCs were used as a source of manganese. Elemental analysis of tissues of various organs of potato demonstrated that manganese was detected after 28 days of growing on such a medium.

Earlier, we published an article devoted to the influence of the NC on the biometric and biochemical characteristics of soybean seedlings [5]. We discovered that nanopriming of soybean seeds infected with the phytopathogenic bacterium *Pcc* substantially reduced the negative impact of the phytopathogen on germination, biometric and biochemical parameters of soybean seedlings. It was shown that the NC nanopriming reduced the levels of primary (DC) and secondary (MDA) lipid peroxidation products. This result may be associated with variations in the FA composition of soybean seedlings. It is known that the interaction of phytopathogenic microorganisms with plants causes substantial changes in the functional state of the latter, which is reflected primarily at the molecular level, while including the FA composition [42]. In the lipid pool of soybean seedlings (control group), 18 FAs were identified, which contain from 12 to 23 carbon atoms. Meanwhile, the lipid composition of the infected samples has been characterized by the presence of 20 kinds of FAs. This suggests that in order to respond to biotic influences, plants need, albeit to some small extent, some enrichment of the lipid composition of their membranes with branched-chain FAs (i.e., C16:0-i; C17:0-i; C20:1(n-11)). The treatment with nanopriming shows the presence of 17 kinds of FAs. Perhaps this effect may be directly related to the antimicrobial effect of NC on *Pcc*, which we demonstrated earlier [5]. NCs could disinfect soybean seeds from the microflora that the seeds were saturated with. The greatest diversity of the FA composition was observed in the “*Pcc* + NC” treatment. There were FAs, which were present only in the infection treatment and only during nanopriming. This suggests a redistribution of FA within the cell membranes.

The biochemical composition of seedlings is determined by the reserve substances stored in the seed tissues [43]. Soybean seed germination rates depend on many biochemical processes such as triacylglycerol hydrolysis and conversion of fats into sugars [44]. In soybean seeds, the complete FA profile is not usually analyzed, but analyzed is the content of the main FAs of soybean oil. Soybean oil is known to contain three USFAs, i.e., OA, LA, and LNA.

PUFAs constitute the largest part (46–78%) of the total FAs in various legumes, including soybeans [45]. The details of SFA biosynthesis remain mostly undiscovered, and there exists a scarcity of research on this subject [46]. For commercial soybeans, the desired content of PA in seeds is 10%, SA C18:0—4%, OA C18:1—18%, LNA C18:2—55%, LA C18:3—13% [28]. An elevation in the content of USFAs (LA, OA, LNA) contributes to the nutritional value of soybean oil [28]. In our investigation, those FAs also prevailed, although they were analyzed in seedlings, as opposed to seeds, as described above. For example, the content of LA 18:2 (n-6) was 32.8%, PA 16:0—28%, LNA C18:3 (n-3)—18.6%, SA 18:0—7.5% and oleic FA C18:1 (n-9)—4.1%.

It is known from the literature that the FA profile in soybeans is associated with such physical properties as seed color and weight [47]. For example, small seeds were found to contain higher levels of LNA (8.53%), PA (44.23%) and LA (55.06%). A clear dependence of the color of the seeds on the FA profile was also revealed. The highest content of PA was in black seeds, a slightly smaller content in yellow seeds, and obviously smaller in brown seeds. The content of LA was 55.07% in black seeds, up to 54.62% in brown seeds and 54.01% in yellow seeds. In terms of the content of LNA, black seeds ranked as the highest, brown seeds were in the middle, and yellow seeds had the lowest concentration of this kind of FA [47]. In our investigation, yellow seeds of the same size were used. It has been shown that the level of USFAs in soybean flour decreases when stored due to hydrolysis [48].

Noteworthily, the overall contents of both SFAs (41–44%) and USFAs (55–58%) in all the studied treatments do not differ substantially from the control samples, except for the treatment with the seeds infected with *Pcc*. In the present investigation, a decrease in the level of unsaturation was observed in the case of nanopriming of uninfected seeds, and an increase in the control level in the case of nanopriming of seeds infected with *Pcc*. Under infection, the content of USFAs in the lipids of soybean seedlings rose by 10% compared to the control level and amounted to 69%. It is known that regulation of the FA unsaturation level in cellular membranes serves as one of the mechanisms through which plants adapt to environmental stressors, while including both biotic and abiotic factors [49]. In this connection, an elevation in the DC content (primary products of lipid peroxidation) may take place, because this is the result of modification of the double-bond arrangement in PUFAs during free radical oxidation of lipids [18]. Earlier, we observed an increase in the DC content in the roots of soybean seedlings with *Pcc* biopriming. Furthermore, a decrease in the DC level was discovered in nanopriming and infection in combination with nanopriming [5]. The PUFA content in these treatments was lower than in (or equal to) the control values.

In most plants, there are three types of main USFAs: OA, LA and α-LNA. These simple compounds play many key roles in plants. The content of these FAs also represents economically important characteristics of oilseed crops [50]. As shown in our investigation, soybean seedlings do not represent any exception, and the main representatives of USFAs are also OA, LA and LNA. The content of OA grew substantially in the NC and infection + NC treatments

In these treatments, the amount of LA was lowered. Infection of the seeds led to an increase in LA content in seedlings. Such variations in the composition of FA may be some part of a specific response mechanism of soybean seedlings to these kinds of treatment. It has been shown that the OA content in soybean seeds may vary depending on the influence of internal and external factors during plant cultivation in the field—genotype, location, weather conditions [29]. OA is involved in signaling during plant defense against pathogens [51]. In our investigation, OA was substantially higher in the treatment with nanopriming, and the content of very-long-chain FAs (VLCFAs) grew. This suggests that nanopriming probably promotes seedling growth and development. OA and LA play many important roles in plant cells, and their roles are associated with responses to both biotic and abiotic stresses. In addition to functioning as membrane components and serving as carbon and energy reserves in triacylglycerols, C18 FAs serve as internal antioxidants, the initial components of various biologically active substances (i.e., jasmonic acid (JA) identified as a stress-related hormone), and reserves for the synthesis of such components of extracellular lipophilic cell wall barrier as cutin and suberin. Moreover, C18 FAs directly play a regulatory role in the plant stress response. For example, C18:1 is involved in the interaction of salicylic acid and JA signaling pathways that prevent penetration of pathogens [50].

In our investigation, the SFAs of soybean seedling lipids were mainly represented by PA (C16:0) and SA (C18:0). No substantial variations in the content of SA were found. The content of PA in seedlings decreased under seed infection. PA is the first FA produced during their synthesis and is the precursor to longer FAs. It has previously been shown that the most tightly held membrane lipids are usually enriched in SFAs, in particular, PA (16:0) [52].

Very-long-chain FAs (i.e., FAs containing over 20 carbon atoms; VLCFAs) are known to play substantial physiological and structural roles in plants [53]. Plant epidermal cells utilize VLCFAs to create cuticular waxes, substances vital for numerous plant–environment relationships. Essential for nutrient homeostasis and plant resilience to adverse conditions, the root suberin barrier is a primary constituent of VLCFAs. Such important lipids as phosphatidylserine, phosphatidylethanolamine, and sphingolipids, which are necessary for maintaining the stability and proper functioning of cell membranes, contain VLCFA. These lipids are involved in the organization of membrane domains, interlayer communications, and intercellular signaling [43]. It is expedient to note that the total content of VLCFAs (C20:0, C20:1(n-11), C22:0 and C23:0) in soybean seedlings grows under nanopriming, as well as under infection treatment in combination with nanopriming, compared to the control.

The effects of NP on the FA profile in soybean tissues have been demonstrated at both the vegetative stage and the seedling stage. Soaking of soybean seeds in a solution of Cu-chitosan NPs for 10 min provoked reduction in the disease occurrence caused by *Pseudomonas savastanoi* pv. *glycinea* and *Curtobacterium flaccumfaciens* pv. *flaccumfaciens*, and increase in the germination of seedlings [54]. Nanopriming of soybean seeds pre-infected with pathogens causing bacterial blight, rust, and soybean wilt, with the use of Cu-chitosan NPs, showed substantial reduction in both the disease incidence in seedlings and the rate of disease development. However, the mechanisms of the protective effects have not been demonstrated [54]. Changes in the FA profile (with respect to PA, OA, LA and LNA), variations in the content of some mineral elements (Fe, Mg, Ca and P) and chlorophylls were similar to those of lipid and protein levels, in the sense that all the parameters measured elevated with the growth in the concentration of ferrous nano-oxide particles (within the range of 0–0.75 g/L.). However, a decrease in all the parameters was observed for the concentration range of 0.75–1 g/L. Nevertheless, application of ferrous nano-oxide particles within the range of 0.75–1 g/L produced the largest impact on the nutrient composition of soybean seeds [55].

The effect of Se NPs on the FA profile has been shown not only for soybeans but also for other agricultural crops. Ahmad et al. [56] assumed that Se NPs stimulate biosynthesis of USFAs (such as OA, LA and α-LA). These researchers showed that nanopriming of sesame seeds with Se NPs caused an increase in antioxidant enzyme activity in seedling tissues. Elevated levels of sesamin, sesamol, tocopherols and USFAs (both in healthy plants and under biotic stress influences) have also been registered [56].

### 3.3. Expression of Desaturase Genes in Soybean Seedling Tissues Under Biotic Stress and Nanopriming

The observed changes in the FA composition are believed to result from the activity of enzymes involved in lipid synthesis and conversion: enzymatic desaturation of FAs [24]. The desaturation ratios given in Table 1 are the indices, which indirectly reflect the activity of desaturase enzymes that catalyze the introduction of double bonds into the carbon chains of FAs. SDR (stearoyl desaturation ratio) is the parameter that indirectly reflects the activity of ω9-desaturases, which add double bonds to the position of ω9. ODR (oleoyl desaturation ratio) is the parameter that indirectly characterizes the activity of ω6-desaturases, which are responsible for catalyzing the addition of double bonds at the position of ω6. LDR (linoleoyl-desaturation ratio) is the indicator indirectly associated with the activity of ω3-desaturases involved in the metabolism of FAs with triple bonds [57]. So, SDR, ODR, and LDR provide indirect information about the specific activity of definite desaturases [58,59]. It is known that FA desaturases are responsible for the conversion of SFAs into unsaturated ones [50]. The activity of FA desaturases influences the phase state (fluidity) of membranes, which is considered an important determinant of cellular metabolism. The data presented in Table 1 demonstrate that the activity of acyl-lipid membrane ω9-desaturases increased substantially in all the examined treatments compared to the control, which is reflected in the content of OA, so, it has also grown. The activity of ω6-desaturases decreased, resulting in slight growth in the LA content in the treatment with NC without infection and in the treatment with NC of *Pcc*-infected plants. An insubstantial decrease in the parameter related to the activity of ω3-desaturases and a reduction of the content of LNA in the seedling lipids have also been revealed.

The catalysis of LA is conducted with the aid of ω6-desaturase (FAD2), which has two distinct isoforms: the first isoform (FAD2-1) is present exclusively in seeds, while the second one (FAD2-2) is both in seeds and in vegetative tissues [28]. LNA is produced in the process of desaturation of 18:2-esterified phosphatidylcholine, a process which involves FAD3A, FAD3B, and FAD3C [28]. In the case of soybean plants, it has been shown that the soybean aphid (*Aphis glycines* Matsumura) causes disturbances in the FA desaturation pathway (C16:0, C18:0, C18:1, C18:2 and C18:3), probably by reducing the activity of FAD2 and FAD6, which leads to a decrease in the content of PUFAs in soybean leaves and in root tissues [60]. Therefore, FA desaturases are considered key components in plant lipid metabolism. Furthermore, these regulate plant–pathogen interactions [61].

A family of enzymes known as acyl-CoA desaturases catalyzes desaturation of FAs. Stearoyl-acyl carrier protein desaturase (SAD) is an important enzyme participating in this mechanism. In higher plants, SAD catalyzes the first step of desaturation (of SA), leading to the formation of OA, which can subsequently be converted into LA and α-LNA. Consequently, SAD is crucial from the viewpoint of its influence on the overall USFA content. This family of enzymes has been found only in plastids of higher plants (unlike other desaturases, such as acyl-lipid desaturases and acyl-CoA desaturases). According to the literature data, this enzyme is so active that almost all newly formed stearoyl-ACPs are quickly converted into oleoyl-ACP [62]. The FA biosynthesis 2 (FAB2) desaturase, a FAD found in the plastid matrix, plays a crucial role in the formation of a double bond at the Δ9 position, and it facilitates the conversion of SA into OA [63]. It has previously been shown that soybean has four genes of the FAB2 subfamily, i.e., three *GmFAB2* genes and one *GmSACPD* gene. *GmFAB2.1* and *GmFAB2.2* are grouped, while *GmFAB2.3* is in a separate branch (along with *GmSACPD*). Furthermore, the expression of *GmFADs* was substantially altered for soybean seeds infected with the seed rot pathogen *Fusarium fujikuroi*. In particular, the *GmFAB2.1/2.2*, *GmFAD3.3/3-2B/7-1/8-2*, and *GmFAD2.3/2.5* genes exhibit different temporal expression patterns in resistant ND25 and susceptible CX12 cultivars of soybean, indicating their potential role in resistance to *F. fujikuroi* infection [61]. In this regard, we investigated the expression of two *FAB2* subfamily genes: *GmFAB2.1* and *GmSACPD*. As a result, we revealed that the expression level of the *GmFAB2.1* gene transcripts in the examined treatments did not differ substantially from the control. Nevertheless, in the treatments of seeds infected with *Pcc* and infected with *Pcc* followed by the nanopriming with Se/AG NC, an observed tendency towards an elevation in the expression of this gene was registered. Furthermore, a substantial elevation in the content of OA was observed in the treatment of seeds with the Se/AG NC nanopriming alone and in the treatment of seeds infected with *Pcc* followed by the Se/AG NC nanopriming. Earlier, the FA composition in leaves and seeds of *Arabidopsis thaliana* mutants *fab2-1*, *fab2-2*, and *fab2-3* was analyzed. This analysis shows that the content of SA (18:0) in the leaves of *fab2* mutants is 9–14.7-fold higher than in the wild type, and the content of 18:0 FAs has grown by 2.7–3.1-times in *fab2* mutants. In the present investigation, another gene of the *FAB2* subfamily, *GmSACPD*, was studied. In the work of Li et al., 2025, it was demonstrated that the expression of *GmFAB2.3* and *GmSACPD* was suppressed at the early stage of *F. fujikuroi* infection (6 h after inoculation) in both the resistant variety ND25 and in the susceptible variety CX12 of soybean [61]. However, their expression recovered faster and reached a peak in 12 h after infection in the resistant variety, as opposed to the susceptible one. Noteworthily, these genes of the same family demonstrated a different level of expression. There are five genes that constitute the soybean stearoyl-acyl carrier protein desaturase (*GmSACPD*) gene family. A curious observation in soybean is the differential impact of *GmSACPD* gene mutations; those affecting *GmSACPD-C* genes resulted in adverse influences on plant growth or nodule function, whereas de novo mutations in *GmSACPD-A*, *GmSACPD-B*, and *GmSACPD-D* genes did not exhibit such consequences. The prevalence of *GmSACPD-C* transcripts across different soybean tissues makes this discovery expected. According to Lakhssassi et al. (2020), nodule development was unaffected in *GmSACPD-A*, *GmSACPD-B*, and *GmSACPD-D* gene mutants because these genes exhibited substantially lower expression in nodules than *GmSACPD-C* [64]. Across all examined plant tissues, such as leaves, roots, seeds, and nodules, the *GmSACPD-C* gene exhibited the most robust expression, whereas *GmSACPD-D* showed the least. However, the SA content was observed to increase by a factor of 2 to 3 when mutations took place in the *GmSACPD-A*, *GmSACPD-B*, and *GmSACPD-D* genes. So, it was revealed that even though *GmSACPD-A*, *GmSACPD-B*, and *GmSACPD-D* were expressed at low levels, these played an important role in elevating 18:1-acyl carrier protein (ACP) in soybean seeds. Another earlier investigation showed that membrane-associated desaturase *GmSACPD* in soybean is involved in resistance to *Pseudomonas syringae* pv. *glycinea* [51]. Taking the above information into account, it may be assumed that the identified expression levels of *GmFAB2.1* and *GmSACPD* genes determine the content of SA and OA in both the control and the treatments, while maintaining these at definite levels necessary for further desaturation. Furthermore, in the examined treatments, membrane-bound acyl-lipid desaturase may be involved in the change in the OA content. This is because the activity values of this desaturase (SDR) reflect the level of OA.

The membrane-bound FAD gene family plays a vital role in processes of plant growth, development, and in responses to stress factors [65]. Membrane-bound FAD proteins (ADS, SLD, DES, FAD6, FAD2, and FAD3/FAD7/8) have been identified in soybean seeds. GmFAD5 and GmFAD3, as well as their isozymes GmFAD7/FAD8, have been revealed in both the endoplasmic reticulum and chloroplasts. The *FAD3*/*FAD7*/*FAD8* subfamily contains eight soybean FAD genes (four *GmFAD3*, two *GmFAD7*, and two *GmFAD8*), which encode the corresponding microsomal and plastid ω3-desaturases [61]. *FAD8-2* is a membrane-bound ω3-desaturase [66]. The *FAD8* gene encodes an enzyme responsible for transforming diene FAs into triene FAs, and this enzyme is crucial for the production of α-LNA [67]. In our investigation, an increase in the expression level of the *GmFAD8-2* gene was observed after infection of seeds with *Pcc*. In this treatment, the observed expression changes could lead to an increase in LNA content. In other treatments, a decrease in the content of this FA compared to the control was observed. At the same time, the activity of membrane-bound LDR, which was assessed indirectly, insubstantially decreased in the experimental treatments. It was noted that most genes in the susceptible soybean variety CX12 were substantially activated at an earlier stage of infection (12 h after inoculation), while for the resistant variety ND25, these were activated only 48 h after infection. Specifically, a six-fold increase in the relative expression of six genes—*GmFAD8.2*, *GmFAD3.3*, *GmFAD7-1*, *GmFAB2.2*, *GmFAD2.3*, and *GmFAD2.5*—was observed 48 h after the inoculation (when compared to the treatment before the inoculation) [61]. The *A. thaliana fad7*/*fad8* double mutant exhibited reduced accumulation of triene FAs in chloroplasts and elevated the sensitivity to *Pseudomonas syringae* pv. *tomato* DC3000 [68]. It has previously been shown that high expression of genes (such as *FAD3*, *FAD7*, and *FAD8*) substantially elevates the content of LNA. Furthermore, a high expression level of *FAD8* may play a crucial role in the formation of PUFAs [67,69]. In our investigation, in the treatment of seeds infected with *Pcc*, we registered an increase in the content of PUFA and USFA in seedlings.

### 3.4. ABA Content in Soybean Seedling Tissues Under Biotic Stress and Nanopriming

It is known that the expression of desaturase genes is regulated by a complex of external and internal factors, in particular, stress factors and hormones [70,71]. ABA is the key phytohormone regulating plant growth and stress responses. ABA influences the activity of enzymes, which interact with Fas, and their biosynthesis in seeds, while intensifying the accumulation of mono-USFAs in seeds. ABA content has been studied in soybean seedlings under salinity [72] and osmotic stress [73] as an indicator of abiotic stress. ABA under biotic stress is also of interest. It is reasonable to assume that the increase in endogenous ABA content results from the intensification of its biosynthesis and decrease in its metabolism. Consequently, desaturases may be considered vital components of plant signaling and defense mechanisms, while aiding in adaptation to biotic and abiotic stress factors. Experimental findings conclusively supported the idea that phytohormones regulate the desaturase gene expression, as shown by studies of the *A. thaliana FAD2* gene promoter. It was shown that *FAD2* gene expression was regulated by 24-epibrassinolide, ABA, and salicylic acid, and this regulation was tissue-specific and dose-dependent [74]. In our investigation, we found that ABA levels were elevated after infection. However, in the infected plants nanoprimed with the Se/AG NC, the hormone content remained at the control level. This result indicates the role of the NC in reductions in the stress load on the plant organism. This result was also confirmed by the results of FA analysis in soybean seedling tissues. The role of ABA in pathogenesis is often related to the stimulation of callose synthesis, which provides protection against the penetration of bacteria and other pathogens [75]. However, excess callose can negatively influence the symplastic transport of substances and, consequently, plant growth. Therefore, the absence of sharp growth in ABA levels in infected plants primed with the NC may be important in maintaining plant growth under the conditions of biotic stress.

### 3.5. Possible Mechanisms of Influence of Se NC on the Phytopathosystem

**Figure 6:**
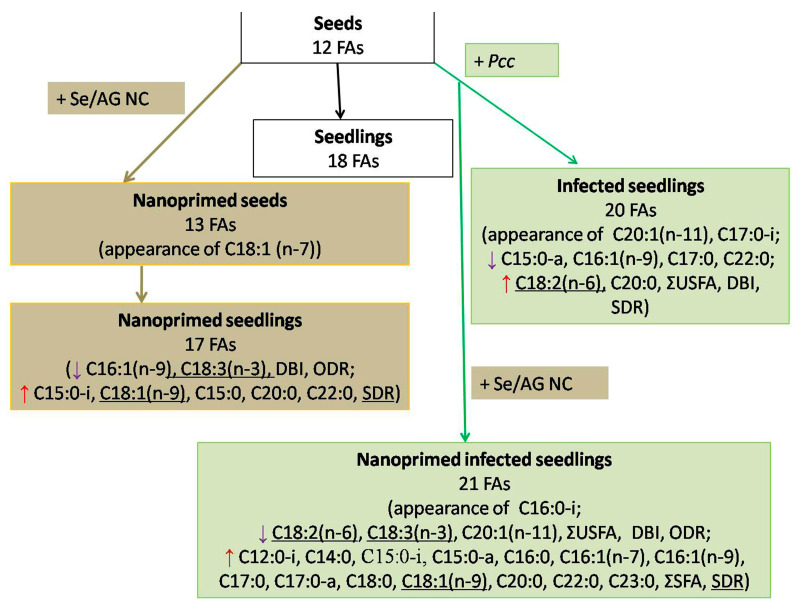
Schematic representation of changes in the FA profile in soybean seed and seedling tissues under the influence of *Pcc* infection and nanopriming. The purple arrow indicates a decrease, and the red arrow indicates an increase in the level.

Priming is known to facilitate seed resource utilization and induce changes in physiological and biochemical parameters [5]. In our experiments, no substantial changes were found in the essential FAs of soybean seed lipids immediately after nanopriming. Meanwhile, a definite influence of NC on the FA profile of lipids was revealed in the tissues of soybean seedlings (Figure 6).

It has been demonstrated that nanopriming in seedling tissues of both *Pcc*-infected and control plants changes the proportions of C18 USFA: OA, LA, and LNA. OA, LA and α-LNA are USFAs, which come to the fore as a kind of general defense system against various biotic and abiotic stresses in plants. These are also the substances that can influence the oxidative stress [76]. C18UFAs are intrinsic antioxidants, i.e., they can directly react with ROS and, so, neutralize ROS. And their oxidation leads to the formation of various oxylipins, represented, for example, by the stress hormone JA, which, in turn, modulates ROS levels and signaling pathways. ATPase activity appears to correlate with the DBI [76]. Elevated OA levels (observed in our experiments in cases of nanopriming) in cell membranes are probably associated with the increased plant resistance to stress. These molecular adaptations represent the result of complex gene regulations aimed at protecting cellular structures and maintaining metabolic processes under adverse conditions, which are critical in maintaining membrane fluidity [77,78]. Another investigation proved that Se NPs (40 mg/L) intensified the formation of various USFAs in sesame plant tissues (*Sésamum índicum* L.). The authors concluded that Se NPs stimulated the biosynthesis of USFAs such as OA, LA, and α-LNA [56]. In our results, the rise in the OA level under nanopriming was accompanied with an increase in the SDR index (this index indirectly indicates the activity of the enzymes involved in the conversion of SA to OA). This may suggest enhanced mobilization and modification of the plant’s lipid pool aimed at adaptation to the changing conditions.

The difference in the influence of nanopriming on the FA profiles of seeds and seedlings may be due to the varied intensity of the endophytic bacterial load on plants, which may be modulated by NC. We earlier demonstrated that *Bacillus* spp. bacteria might be revealed in the tissues of soybean seedlings [79]. These bacteria produced a negative effect on potato plants artificially infected *in vitro*. In the seeds, these bacteria were most likely present in the form of spores. During seed germination, when the conditions inside the seed became more favorable for bacteria (with optimal temperature and humidity), and the enzymatic destruction of complex storage substances into those more easily decomposed by bacteria, bacteria of the *Bacillus* genus emerged from the spore stage, while generating some additional stress for the plant organism. In one of our earlier investigations, we demonstrated that the probes of seed nanopriming with NC can cause a decrease in the number of bacteria of the *Bacillus* spp. in the endomicrobiome of soybean seedlings [79]. It is known that Se NPs possess antioxidant effects [80]. Therefore, the beneficial effect of NCs may be directly related to the suppression of the endogenous microbiota, as well as to a decrease in the intensity of oxidative stress in plant tissues caused by these bacteria. Being treated with optimal concentration of Se NPs, seeds are capable of neutralizing ROS, enhancing antioxidant activity, reducing the oxidative stress, and elevating resistance to stress factors. This beneficial effect extends not only to the seed germination and seedling growth stages. It also persists throughout the entire life cycle of any plant, with this fact being described in the literature [13].

So, we assume that the probable mechanisms of the influence of NC on the plant organism are, taking into account previously obtained and published data on the biological effect of Se/AG NC on plant–microbial relationships, as follows: (i) reduction in oxidative stress in plant tissues; (ii) activation of proteins involved in pathogenesis; (iii) direct antibacterial effect on phytopathogenic bacteria; (iv) influence on the hormonal status of the plant.

## 4. Materials and Methods

### 4.1. Plant Material and Bacteria Strains

**Figure 7:**
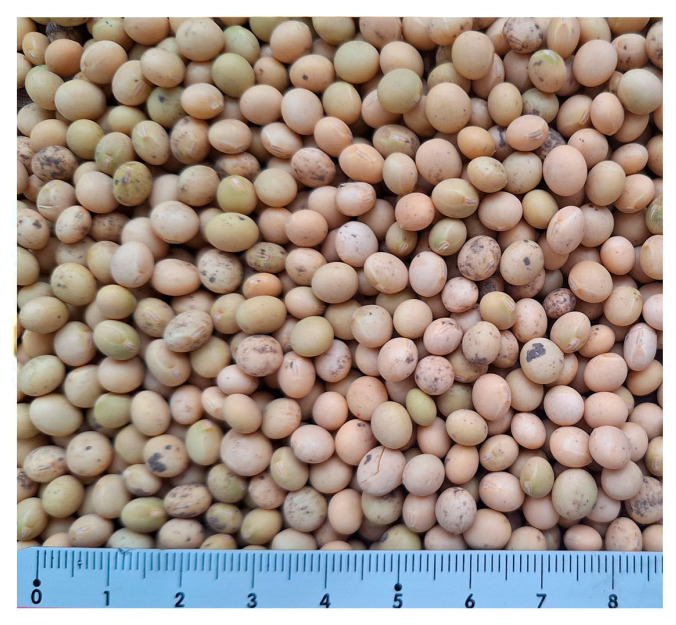
A photo of soybean seeds of the variety “Sayana”.

In our investigation, we used *Glycine max* (L.) seedlings of the variety “Sayana”, known for its superior tolerance to cold during germination and its ability to produce larger yields under long-day conditions with insufficient heat [81]. A photo of soybean seeds is shown in Figure 7.

Our investigation used the Gram-negative bacterium *Pcc*, strain VKM B-1274, obtained from the All-Russian Collection of Microorganisms (VKM IBPM RAS). The bacteria were grown in meat-peptone broth (MPB). The scale bar shown below in Figure 7 represents centimeters. The bacterial suspension of *Pcc* was cultivated to high titers, such as 10^8^ PFU/mL. We deliberately chose a high titer of bacterial cells in order to clearly see the effect of bacterial influence and the effect of leveling this influence with aid of the NC used.

### 4.2. Nanocomposite

Se/AG NC was synthesized with the aid of the following method [32]. A solution of 1 g of AG (on the basis of raw materials obtained from Siberian larch *Lárix sibírica* (bioflavonoid content being ~5% [31])) in 6 mL of water, with mass of 20 μL of 25% aqueous ammonia solution, was added (being accompanied by vigorous stirring), while bringing the reaction mixture to a neutral medium (pH = 7.37). The final reaction mixture was stirred for 20 min. Next, 0.1938 mM of aqueous solution of selenious acid (1 mL H_2_O and 25 mg H_2_SeO_3_) was introduced (dropwise) into the reaction mixture, and the final reaction mixture was stirred for 40 min.

Next, the mass of ~63 μL of 25% aqueous ammonia solution was added dropwise again (until pH = 7.41), and the mixture underwent further stirring for 30 min. The final reaction mixture was filtered through filter paper and slowly poured with stirring into a threefold excess of isopropyl alcohol. A light-beige NC precipitate was observed. The precipitate was decanted three times in a centrifuge with the addition of 80% isopropyl alcohol and, next, dried in vacuum. The resulting powdered nanocomposite was represented in light beige. Elemental analysis of the Se content in the nanocomposite was conducted on an inductively coupled plasma atomic emission spectrometer iCAP PRO (Thermo Fisher Scientific, Waltham, MA, USA). Optical absorption spectra of aqueous colloidal solutions of the nanocomposite were measured on a UV/visible light optical spectrometer Shimadzu UV-1900 (Shimadzu Corporation, Kyoto, Japan) in the wavelength range of 190–1100 nm. The size distribution of NPs throughout NC in an aqueous colloidal solution was analyzed by dynamic light scattering with the use of a spectrometer PHOTOCOR COMPACT Z (Photocor LLC, Moscow, Russia). As a result, a colloidal aqueous solution of Se/AG NC particles with hydrodynamic dimension of 125–436 nm (Se concentration of 0.000625%) was used for nanopriming soybean seeds.

### 4.3. Experimental Design

Disinfecting, infecting with *Pcc*, nanopriming seeds and germination before sampling were conducted as described in [5].

For the purpose of seed treatment, the concentration of Se/AG NC was 50 µg/mL (3 µg/mL Se), including concentration of 0.000625% Se (6.25 µg/mL) in the final suspension. This concentration was chosen by us experimentally. The phytotoxicity of this concentration and other concentrations of Se/AG NC was studied on potato plants *in vitro* [82]. This is the concentration which makes it possible to suppress the growth of phytopathogenic bacteria and fungi [5,83,84]. At the same time, it does not produce any adverse impact on the growth and development of plants. Furthermore, it even stimulates these processes [17,39,82,85]. The phytotoxicity of this concentration and other concentrations of Se/AG NC was studied on potato plants *in vitro* [86].

The present article is one in a series of research publications devoted to investigations of biological effects of Se/AG NC on cultivated plants. Our work deals with the investigation of biological activity of NC on two crop species: potato and, later, soybean. In the initial stage, our investigations were conducted on potato tubers *in vitro*. In this case, we initially investigated the influence of the Se/AG NC and its precursors, AG and Se-containing compounds. So, as a control for studying the influence of Se/AG NC on the biometric and biochemical characteristics of potato plants *in vitro*, we investigated the effects of NC precursors—AG and selenium oxide [87]. It was discovered that the viability of plants was diminished by the precursor of selenium oxide compared to the approach presuming the Se/AG NC treatment. AG did not influence the growth and development of the plants. In another article, we also investigated the effect of the precursor of the Se/AG NC—sodium bis(2-phenylethyl)diselenophosphinate (BIS)—on viability of potato *in vitro* [39]. It was also found that, in case of usage of Se/AG NC and BIS (in the same concentrations) for treatment of plants, BIS suppressed processes of plant growth and development; meanwhile, the Se/AG NC stimulated all the studied parameters.

Therefore, these results give evidence that the observed effect produced on the biometric and biochemical characteristics of plants is associated with the influence of this nanocomposite but not with its precursors. Therefore, in our later investigations, we did not use the “AG” and “BIS” treatments.

To determine the FA content of soybean seeds, the seeds were disinfected as described above. The control samples were then soaked in water for 30 min, while experimental samples were soaked in Se/AG NC solution. The samples were frozen in liquid nitrogen, and the FA content was then determined.

### 4.4. Determination of the FA Composition of Membrane Lipids in Soybean Seedlings

The average biomass weighing 0.3 g per sample was selected for analysis. A modified technique [88] was used to determine the FA composition of lipids. Parameters, which indirectly indicate the activity of membrane-bound acyl-lipid ω9-, ω6-, and ω3-desaturases, responsible for double-bond formation in the hydrocarbon chains of OA, LA and LNA, respectively, were represented by the ratios of stearoyl- (SDR), oleoyl- (ODR), and linoleyl- (LDR) desaturase ratios according to the following formulas [89,90]: (1) SDR (activity of ω9-desaturases) = (%C18:1)/(%C18:0 + %C18:1), (2) ODR (activity of ω6-desaturases) = (%C18:2 + %C18:3)/(%C18:1 + %C18:2 + %C18:3), (3) LDR (activity of ω3-desaturases) = (%C18:3)/(%C18:2 + %C18:3). The DBI was used as a characteristic of the degree of FA unsaturation.

### 4.5. RNA Isolation and RT-qPCR

RNA was extracted from 5-day-old soybean etiolated plantlets with RNeasy mini kit (QIAGEN, Hilden, Germany). cDNA synthesis and RT-qPCR were conducted according to [91]. The expression of each gene was normalized against the expression of *EF4* gene [92]. RT-qPCR analysis was repeated three times with the independently extracted RNA. In our investigation, we studied the expression of such FAD genes as *GmSACPD*, *GmFAD8-2*, and *GmFAB2.1*. The primers for the soybean desaturase genes were taken from a previously published article [61]. The primers are listed in Table 2.

### 4.6. Determination of Phytohormone Content

ABA was extracted from lyophilized seedling tissues with the use of 70% ethanol (4 °C, overnight). On the next day, the samples were centrifuged; the supernatant was collected, and the alcohol was removed under a stream of air. Hormone purification was conducted via ether extraction, as described, e.g., in [93]. Briefly speaking, the hormone was transferred from the acidified (pH 2–3) aqueous residue (acidified with hydrochloric acid) first into the ether phase, then into an alkaline sodium bicarbonate solution, and, finally, back into ether following re-acidification. At each successive step, the volume of extractant was reduced, thereby increasing the selectivity of hormone extraction [94]. In the final stage, freshly prepared diazomethane was added to the ether to methylate ABA.

The ABA content was determined by enzyme-linked immunosorbent assay (ELISA) using specific rabbit polyclonal antibodies raised against this hormone [93]. The wells of polystyrene microplates were coated with an ABA–ovalbumin conjugate. After 2 h of incubation at 37 °C, the solution was washed off with phosphate-buffered saline (PBS) containing 0.05% Tween 20. Subsequently, either the sample or a ten-fold serial dilution of the hormone standard (for constructing the calibration curve) was added to the wells. Next, a solution of primary antibodies was added to all wells, followed by incubation for 1 h at 37 °C. Unbound antibodies were removed by washing, and horseradish peroxidase (HRP)-conjugated goat anti-rabbit secondary antibodies were applied. After 1 h of incubation, the amount of bound secondary antibodies was quantified by a colorimetric reaction using o-phenylenediamine (OPD) dissolved in phosphate–citrate buffer (pH 5.5) supplemented with 0.03% H_2_O_2_. Optical density was measured at 492 nm using a UNIPLAN AIFR-01 microplate spectrophotometer (PIKON, Moscow, Russia).

### 4.7. Statistical Data Analysis

For the purpose of FA analysis of the seeds, 10 soybean seeds were used for each treatment. The normality of data distribution was assessed with the use of the Shapiro–Wilk test. The distribution was found to be non-normal, so median [Q1;Q3] is presented in Table S1. Levels of significance were assessed with the use of the Mann–Whitney U test.

In the interest of constructing a correct experiment, in each treatment, the seeds were grown in three Petri dishes. Each dish contained 10 seeds. A total sample containing seedlings obtained from 5–8 seeds was formed from each dish.

Three independent experiments were conducted to fulfill the FA analysis.

Five independent experiments were conducted to fulfill the molecular–genetic analysis and to determine the ABA content.

When analyzing the results of the FA analysis, the Shapiro–Wilk test was used to determine the normality of the sample. Next, the ANOVA test was used for multiple comparisons, and the Student’s coefficient was used for pairwise comparisons. The obtained results were presented as an arithmetic mean (M), and the spread of values as the standard error (±S.E.) and a median with quartiles for nonparametric data. Differences between experimental data were considered statistically plausible at *p* < 0.05 and *p* < 0.01. The substantial character of differences was determined with the use of the ANOVA test for multiple data comparisons, the Student’s *t*-test for pairwise comparisons and the Shapiro–Wilk test for nonparametric data in SigmaPlot software v.12.5 (SYSTAT Software, Chicago, IL, USA).

## 5. Conclusions

For the first time, this investigation studied the FA composition of soybean seeds of the “Sayana” variety, which is suitable for cultivation in risky farming zones. The FA profile was represented by 12 FAs. Furthermore, the FA composition of seedlings of this soybean variety was analyzed. The results of the investigation show that nanopriming and infection (separately and in combination) lead to variations in the FA profile of soybean seedlings, suggesting that FAs participate in responses to external (or applied) treatments. Infection of the seeds with *Pcc* leads to an increase in USFAs. Nanopriming has little influence on variations in the content of FAs in seedling lipids. However, nanopriming infected seeds has demonstrated an effect in reducing the negative impact of *Pcc*, probably allowing the seedlings to retain membrane microviscosity at the normal level. Variations in FA content in the treatments studied concerned mainly C18 FA and LC-PUFAs. As described above, these compounds are often involved in signaling pathways. Therefore, we put forward an assumption that nanopriming affects these cellular signaling pathways, which are necessarily involved in response to biotic stress. This may explain why the NC suppresses phytopathogens and promotes germination of seeds, while improving some morphological properties of soybean seedlings. The results of the investigation described in the present article show an increase in the ABA content in plant tissues under biotic stress in the phytopathosystem studied, which complements the knowledge about the role of ABA in the response of plants to stress factors of a biotic nature.

## Figures and Tables

**Table 1:** The content of FAs in lipids (%) and the ratios of stearoyl, oleoyl, and linoleoyl desaturation (SDR, ODR and LDR, respectively) in soybean seedlings after infection of soybean seeds with *Pcc* and nanopriming with Se/AG NC. M ± S.D. (*n* = 6). *n* is the number of samples obtained based on two analytical replications in three independent experiments, in which each sample consisted of the material from 5–8 seedlings.

FA	Control	*Pcc*	Se/AG NC	*Pcc* + Se/AG NC
C12:0-i	0.09 ± 0.01	0.07 ± 0.0	0.10 ± 0.01 **	0.10 ± 0.01 ^††^
C14:0	0.51 ± 0.06	0.36 ± 0.04	0.51 ± 0.04	0.45 ± 0.06 *^†^
C15:0-i	0.07 ± 0.01	0.06 ± 0.0	0.11 ± 0.02 *	0.10 ± 0.02 *^†^
C15:0-a	0.31 ± 0.02	0.10 ± 0.1 *	0.25 ± 0.03 *	0.47 ± 0.02 *^†^
C15:0	0.28 ± 0.04	0.20 ± 0.2 *	0.34 ± 0.04 *	0.29 ± 0.03 ^†^
C16:0	28.19 ± 2.31	20.91 ± 1.23 *	29.00 ± 1.55	27.92 ± 4.42 ^††^
C16:0-i	-	-	-	0.21 ± 0.04
C16:1(n-7)	0.18 ± 0.02	0.19 ± 0.02	-	0.25 ± 0.02 *^†^
C16:1(n-9)	0.78 ± 0.09	0.22 ± 0.02 *	0.49 ± 0.01 *	0.40 ± 0.01 *^†^
C17:0-a	0.05 ± 0.01	0.04 ± 0.0	0.06 ± 0.01	0.14 ± 0.01 *^†^
C17:0	1.14 ± 0.19	0.35 ± 0.03 *	1.01 ± 0.14	0.57 ± 0.06 *^†^
C17:0-i	-	0.02 ± 0.0	-	0.06 ± 0.01 ^†^
C18:0	7.44 ± 0.81	6.40 ± 0.59	7.80 ± 0.83	8.24 ± 1.01 ^††^
C18:1(n-9)	4.10 ± 0.74	4.93 ± 0.81	8.22 ± 1.20 *	8.65 ± 1.50 *^†^
C18:1(n-7)	2.20 ± 0.16	2.27 ± 0.12	2.24 ± 0.18	2.46 ± 0.21
C18:2(n-6)	32.83 ± 1.85	40.86 ± 1.44 *	29.57 ± 3.15	30.11 ± 4.33 ^†^
C18:3(n-3)	18.62 ± 2.07	20.66 ± 2.28	15.04 ± 1.18 *	15.09 ± 1.68 *^†^
C20:0	1.55 ± 0.14	1.17 ± 0.14 *	1.91 ± 0.14 *	1.71 ± 0.21 ^†^
C20:1(n-11)	-	0.12 ± 0.01	-	0.09 ± 0.01 ^††^
C22:0	1.23 ± 0.16	0.80 ± 0.05 *	2.88 ± 0.34 *	2.22 ± 0.50 *^†^
C23:0	0.47 ± 0.05	0.29 ± 0.03 *	0.48 ±0.04	0.48 ± 0.04 ^†^
ΣSFA	41.35 ± 3.15	30.76 ± 1.56 *	44.45 ± 1.68	42.96 ± 5.16 ^†^
ΣUSFA	58.65 ± 3.15	69.24 ± 1.56 *	55.55 ± 1.68	57.04 ± 5.16 ^†^
DBI	1.29 ± 0.09	1.51 ± 0.05 *	1.15 ± 0.04 *	1.17 ± 0.12 *^†^
SDR	0.35 ± 0.02	0.43 ± 0.03 *	0.51 ± 0.05 *	0.51 ± 0.05 *^††^
ODR	0.93 ± 0.02	0.93 ± 0.01	0.84 ± 0.03 *	0.84 ± 0.03 *^†^
LDR	0.36 ± 0.02	0.34 ± 0.03	0.34 ± 0.04	0.34 ± 0.02

Note: Substantial differences compared to the control (* *p* < 0.01 and ** *p* < 0.05) or the treatment with *Pcc* infection only (^†^ *p* < 0.01 and ^††^ *p* < 0.05) were based on the ANOVA test for multiple comparisons and the Student’s *t*-test for pairwise comparisons.

**Table 2:** The primers used in the present investigation.

Name	Primer Sequences	Reference
*GmEF4*	F: GATTTCATGTAGCCGTAGCC R: ATTTAAGACATCCCTCCTCAG	[92]
*GmFAB2.1*	F: ACAGGTGCCAGCCTTACT R: TCCATTCCAGACCCAATA	[61]
*GmFAD8-2*	F: TTCCACGGTCAACAAGAC R: CTCACTCCCAATTCCCAC	
*GmSACPD*	F: TCGGACGGTGGAGATTGGAGAAG R: CTCGCTCATCAGCACGCTCTTG	

## Data Availability

All data supporting the conclusions of this article are provided within the article (and its Supplementary Materials).

## Supplementary Materials

The following supporting information can be downloaded at: https://www.mdpi.com/article/10.3390/plants15111647/s1, Table S1: The influence of Se/AG NC on soybean seed FA content. Median [Q1;Q3].

## Abbreviations

The following abbreviations are used in this manuscript:

AG	Arabinogalactan
AOE	antioxidant enzymes
DBI	double bond index
EMF	electromagnetic field
FA	fatty acid
FAD	fatty acid desaturase
FAMEs	methyl esters of FAs
JA	jasmonic acid
LA	linoleic acid
LNA	linolenic acid
LDR	linoleoyl desaturation ratio
MS	Murashige–Skoog medium
OA	oleic acid
ODR	oleoyl desaturation ratio
PA	palmitic acid
*Pcc*	*Pectobacterium carotovorum*
PUFAs	polyunsaturated fatty acids
ROS	reactive oxygen species
SA	stearic acid
Se/AG NC	selenium–arabinogalactan nanocomposite
SDR	stearoyl desaturation ratio
SFA	saturated fatty acids
USFA	unsaturated fatty acids
VLCFAs	very-long-chain fatty acids

## References

[1] Perfileva A.I., Strekalovskaya E.I., Klushina N.V., Gorbenko I.V., Krutovsky K.V. (2025). The causative agent of soft rot in plants, the phytopathogenic bacterium *Pectobacterium carotovorum* subsp. *carotovorum*: A brief description and an overview of methods to control it. Agronomy.

[2] Kang M., Kim S.J., Lee J.Y., Yoon S.R., Kim S.H., Ha J.H. (2018). Inactivation of *Pectobacterium carotovorum* subsp. *carotovorum* on Chinese cabbage (*Brassica rapa* L. subsp. *pekinensis*) by wash treatments with phenolic compounds. LWT.

[3] El-Naggar N.E.A., Bashir S.I., Rabei N.H., Saber W.I.A. (2022). Innovative biosynthesis, artifi cial intelligence-based optimization, and characterization of chitosan nanoparticles by *Streptomyces microflavus* and their inhibitory potential against *Pectobacterium carotovorum*. Sci. Rep..

[4] van der Wolf J.M., Acuña I., De Boer S.H., Brurberg M.B., Cahill G., Charkowski A.O., Coutinho T., Davey T., Dees M.W., Degefu Y., van Gijsegem F., van der Wolf J.M., Toth I.K. (2021). Diseases Caused by *Pectobacterium* and *Dickeya* Species Around the World. Plant Diseases Caused by Dickeya and Pectobacterium Species.

[5] Perfileva A.I., Kharasova A.R., Nozhkina O.A., Sidorov A.V., Graskova I.A., Krutovsky K.V. (2023). Effect of nanopriming with selenium nanocomposites on potato productivity in a field experiment, soybean germination and viability of *Pectobacterium carotovorum*. Horticulturae.

[6] Sun J., Mooney H., Wu W., Tang H., Tong Y., Xu Z., Huang B., Cheng Y., Yang X., Wei D. (2018). Importing food damages domestic environment: Evidence from global soybean trade. Proc. Natl. Acad. Sci. USA.

[7] Gohari G., Spanos A., Ioannou A., Efstathiou I., Panahirad S., Kolbert Z., Fotopoulos V. (2026). Seed priming approaches for climate-resilient agriculture. J. Exp. Bot..

[8] Mitra D., Mondal R., Khoshru B., Shadangi S., Das Mohapatra P.K., Panneerselvam P. (2021). Rhizobacteria mediated seed bio-priming triggers the resistance and plant growth for sustainable crop production. Curr. Res. Microb. Sci..

[9] do Espirito Santo Pereira A., Caixeta Oliveira H., Fernandes Fraceto L., Santaella C. (2021). Nanotechnology potential in seed priming for sustainable agriculture. Nanomaterials.

[10] Khan M.N., Fu C., Li J., Tao Y., Li Y., Hu J., Chen L., Khan Z., Wu H., Li Z. (2023). Seed nanopriming: How do nanomaterials improve seed tolerance to salinity and drought?. Chemosphere.

[11] Ulhassan Z., Yang S., He D., Khan A.R., Salam A., Azhar W., Muhammad S., Ali S., Hamid Y., Khan I. (2023). Seed priming with nano-silica effectively ameliorates chromium toxicity in *Brassica napus*. J. Hazard. Mater..

[12] Donia D.T., Carbone M. (2023). Seed priming with zinc oxide nanoparticles to enhance crop tolerance to environmental stresses. Int. J. Mol. Sci..

[13] Yang L., Zhang L., Zhang Q., Wei J., Zhao X., Zheng Z., Chen B., Xu Z. (2024). Nanopriming boost seed vigor: Deeper insights into the effect mechanism. Plant Physiol. Biochem..

[14] Ulhassan Z., Ali S., Kaleem Z., Shahbaz H., He D., Khan A.R., Salam A., Hamid Y., Sheteiwy M.S., Zhou W. (2025). Effects of nanosilica priming on rapeseed (*Brassica napus*) tolerance to cadmium and arsenic stress by regulating cellular metabolism and antioxidant defense. J. Agric. Food Chem..

[15] Srivastava S., Tyagi R., Sharma S. (2023). Seed biopriming as a promising approach for stress tolerance and enhancement of crop productivity: A review. J. Sci. Food Agric..

[16] Cembrowska-Lech D., Rybak K. (2023). Nanopriming of barley seeds-a shotgun approach to improve germination under salt stress conditions by regulating of reactive oxygen species. Plants.

[17] Dyall S.C., Balas L., Bazan N.G., Brenna J.T., Chiang N., da Costa Souza F., Dalli J., Durand T., Galano J.M., Lein P.J. (2022). Polyunsaturated fatty acids and fatty acid-derived lipid mediators: Recent advances in the understanding of their biosynthesis, structures, and functions. Prog. Lipid Res..

[18] Sidorov R.A., Starikov A.Y., Sinetova M.A., Guilmisarian E.V., Los D.A. (2024). Identification of conjugated dienes of fatty acids in *Vischeria* sp. IPPAS C-70 under oxidative stress. Int. J. Mol. Sci..

[19] Singh P., Arif Y., Miszczuk E., Bajguz A., Hayat S. (2022). Specific roles of lipoxygenases in development and responses to stress in plants. Plants.

[20] Rizzo G., Baroni L., Lombardo M. (2023). Promising sources of plant-derived polyunsaturated fatty acids: A narrative review. Int. J. Environ. Res. Public Health.

[21] Christie W.W., Harwood J.L. (2020). Oxidation of polyunsaturated fatty acids to produce lipid mediators. Essays Biochem..

[22] Harwood J.L. (2023). Polyunsaturated fatty acids: Conversion to lipid mediators, roles in inflammatory diseases and dietary sources. Int. J. Mol. Sci..

[23] Li M., Yu G., Cao C., Liu P. (2021). Metabolism, signaling, and transport of jasmonates. Plant Commun..

[24] Nguyen Q.T., Kisiala A., Andreas P., Neil Emery R.J., Narine S. (2016). Soybean seed development: Fatty acid and phytohormone metabolism and their interactions. Curr. Genom..

[25] Niu Y., Xiang Y. (2018). An Overview of biomembrane functions in plant responses to high-temperature stress. Front. Plant Sci..

[26] de Carvalho C.C.C.R., Caramujo M.J. (2018). The various roles of fatty acids. Molecules.

[27] Zhang S., Feng H., Agyenim-Boateng K.G., Zhang S., Gu Y., Qi J., Feng Y., Li Y., Ma C., Liu Y. (2025). High resolution QTL mapping and candidate gene mining for seed oil content and fatty acid composition in soybean. BMC Plant Biol..

[28] Kumar R., Mulkey S., Shelake R.M., Combs-Giroir R., Mukherjee T., Allen D.K., Clemente T.E., Stacey M.G., Lorenz A.J., Stupar R.M. (2025). Targets and strategies to design soybean seed composition traits. Plant Genome.

[29] Bewick P., Forstner P., Zhang B., Collakova E. (2025). Identification of novel candidate genes for regulating oil composition in soybean seeds under environmental stresses. Front. Plant Sci..

[30] Kaushal C., Sachdev M., Parekh M., Gowrishankar H., Jain M., Sankaranarayanan S., Pathak B. (2025). Transcriptional engineering for value enhancement of oilseed crops: A forward perspective. Front. Genome Ed..

[31] Sukhov B.G., Pogodaeva N.N., Kuznetsov S.V., Kupriyanovich Y.N., Yurinova G.V., Selivanova D.S., Pristavka A.A., Dzhioev Y.P., Popkova S.M., Rakova E.B. (2014). Prebiotic effect of native noncovalent arabinogalactan—Flavonoid conjugates on bifidobacteria. Russ. Chem. Bull..

[32] Sukhov B.G., Ganenko T.V., Pogodaeva N.N., Kuznetsov S.V., Silkin I.I., Lozovskaya E.A., Shurygin M.G., Shurygina I.A., Trofimov B.A. (2017). An Agent Possessing Antitumor Activity Based on Nanocomposites of Arabinogalactan with Selenium, and Methods for Producing such Nanobiocomposites.

[33] Gogoi K., Gogoi H., Borgohain M., Saikia R., Chikkaputtaiah C., Hiremath S., Basu U. (2024). The molecular dynamics between reactive oxygen species (ROS), reactive nitrogen species (RNS) and phytohormones in plant’s response to biotic stress. Plant Cell Rep..

[34] Khutsishvili S.S., Perfileva A.I., Kon’kova T.V., Lobanova N.A., Sadykov E.K., Sukhov B.G. (2024). Copper-containing bionanocomposites based on natural raw arabinogalactan as effective vegetation stimulators and agents against phytopathogens. Polymers.

[35] Abdelsalam A., El-Sayed H., Hamama H.M., Morad M.Y., Aloufi A.S., Abd El-Hameed R.M. (2023). Biogenic selenium nanoparticles: Anticancer, antimicrobial, insecticidal properties and their impact on soybean (*Glycine max* L.) seed germination and seedling growth. Biology.

[36] Xiong Y., Xiang X., Xiao C., Zhang N., Cheng H., Rao S., Cheng S., Li L. (2023). Illumina RNA and SMRT sequencing reveals the mechanism of uptake and transformation of selenium nanoparticles in soybean seedlings. Plants.

[37] Raina S., Sheikh Z.N., Bakshi P., Alamri S., Siddiqui M.H., Islam Rather K.U., Ashraf K. (2025). Functional evaluation of cis-vaccenic acid in cordia dichotoma with tissue-specific biochemical insights. Sci. Rep..

[38] Jacome-Sosa M., Vacca C., Mangat R., Diane A., Nelson R.C., Reaney M.J., Shen J., Curtis J.M., Vine D.F., Field C.J. (2016). Vaccenic acid suppresses intestinal inflammation by increasing anandamide and related N-acylethanolamines in the JCR:LA-cp rat. J. Lipid Res..

[39] Perfileva A.I., Nozhkina O.A., Ganenko T.V., Graskova I.A., Sukhov B.G., Artem’ev A.V., Trofimov B.A., Krutovsky K.V. (2021). Selenium nanocomposites in natural matrices as potato recovery agent. Int. J. Mol. Sci..

[40] Nozhkina O.A., Perfileva A.I., Graskova I.A., Nurminsky V.N., Klimenkov I.V., Dyakova A.V., Ganenko T.V., Borodina T.N., Aleksandrova G.P., Sukhov B.G. (2019). The biological activity of a selenium nanocomposite encapsulated in carrageenan macromolecules with respect to ring rot pathogenesis of potato plants. Nanotechnol. Russ..

[41] Khutsishvili S.S., Perfileva A.I., Nozhkina O.A., Ganenko T.V., Krutovsky K.V. (2021). Novel Nanobiocomposites based on natural polysaccharides as universal trophic low-dose micronutrients. Int. J. Mol. Sci..

[42] Reszczyńska E., Hanaka A. (2020). Lipids Composition in plant membranes. Cell Biochem. Biophys..

[43] Moreira T.B., Shaw R., Luo X., Ganguly O., Kim H.S., Coelho L.G.F., Cheung C.Y.M., Rhys Williams T.C. (2019). A Genome-scale metabolic model of soybean (*Glycine max*) highlights metabolic fluxes in seedlings. Plant Physiol..

[44] Zhou W., Chen F., Zhao S., Yang C., Meng Y., Shuai H., Luo X., Dai Y., Yin H., Du J. (2019). DA-6 promotes germination and seedling establishment from aged soybean seeds by mediating fatty acid metabolism and glycometabolism. J. Exp. Bot..

[45] Sipeniece E., Mišina I., Qian Y., Grygier A., Sobieszczańska N., Sahu P.K., Rudzińska M., Patel K.S., Górnaś P. (2021). Fatty acid profile and squalene, tocopherol, carotenoid, sterol content of seven selected consumed legumes. Plant Foods Hum. Nutr..

[46] Liu J., Dong L., Duan R., Hu L., Zhao Y., Zhang L., Wang X. (2022). Transcriptomic analysis reveals the regulatory networks and hub genes controlling the unsaturated fatty acid contents of developing seed in soybean. Front. Plant Sci..

[47] Abdelghany A.M., Zhang S., Li J., Li B., Sun J. (2025). Seed phenotype and maturity groups as determinants of protein, oil, and fatty acid composition patterns in diverse soybean germplasm. BMC Plant Biol..

[48] Prabakaran M., Lee K.J., An Y., Kwon C., Kim S., Yang Y., Ahmad A., Kim S.H., Chung I.M. (2018). Changes in soybean (*Glycine max* L.) flour fatty-acid content based on storage temperature and duration. Molecules.

[49] Berestovoy M.A., Pavlenko O.S., Goldenkova-Pavlova I.V. (2019). Plant fatty acid desaturases: Role in the life of plants and biotechnological potential. Biol. Bull. Rev..

[50] He M., Qin C.X., Wang X., Ding N.Z. (2020). Plant unsaturated fatty acids: Biosynthesis and regulation. Front. Plant Sci..

[51] Kachroo A., Fu D.Q., Havens W., Navarre D., Kachroo P., Ghabrial S.A. (2008). An oleic acid-mediated pathway induces constitutive defense signaling and enhanced resistance to multiple pathogens in soybean. Mol. Plant Microbe Interact..

[52] Zhukov A.V. (2021). On qualitative composition of membrane lipids in plant cells. Russ. J. Plant Physiol..

[53] Batsale M., Bahammou D., Fouillen L., Mongrand S., Joubès J., Domergue F. (2021). Biosynthesis and functions of very-long-chain fatty acids in the responses of plants to abiotic and biotic stresses. Cells.

[54] Tarakanov R., Shagdarova B., Lyalina T., Zhuikova Y., Il’ina A., Dzhalilov F., Varlamov V. (2023). Protective properties of copper-loaded chitosan nanoparticles against soybean pathogens *Pseudomonas savastanoi* pv. *glycinea* and *Curtobacterium flaccumfaciens* pv. *flaccumfaciens*. Polymers.

[55] Sheykhbaglou R., Sedghi M., Fathi-Achachlouie B. (2018). The effect of ferrous nano-oxide particles on physiological traits and nutritional compounds of soybean (*Glycine max* L.) seed. An. Acad. Bras. Cienc..

[56] Ahmad I., Younas Z., Mashwani Z.U., Raja N.I., Akram A. (2023). Phytomediated selenium nanoparticles improved physio-morphological, antioxidant, and oil bioactive compounds of sesame under induced biotic stress. ACS Omega.

[57] Jaworski J.G., Stumpf P.K. (1974). Fat metabolism in higher plants. properties of a soluble stearyl acyl carrier protein desaturase from maturing *Carthamus tinctorius*. Arch. Biochem. Biophys..

[58] Christie W.W. (1993). Preparation of ester derivatives of fatty acids for chromatographic analysis. Adv. Lipid Method.

[59] Xiao R., Zou Y., Guo X., Li H., Lu H. (2022). Fatty acid desaturases (FADs) modulate multiple lipid metabolism pathways to improve plant resistance. Mol. Biol. Rep..

[60] Kanobe C., McCarville M.T., O’Neal M.E., Tylka G.L., MacIntosh G.C. (2015). Soybean aphid infestation induces changes in fatty acid metabolism in soybean. PLoS ONE.

[61] Li X., Munir M., Zeng W., Sun Z., Chang X., Yang W. (2025). Characterization of fatty acid desaturase gene family in *Glycine max* and their expression patterns in seeds after *Fusarium fujikuroi* infection. Front. Plant Sci..

[62] Hernández M.L., Sicardo M.D., Alfonso M., Martínez-Rivas J.M. (2019). Transcriptional regulation of stearoyl-acyl carrier protein desaturase genes in response to abiotic stresses leads to changes in the unsaturated fatty acids composition of olive mesocarp. Front. Plant Sci..

[63] Xue Y., Chen B.J., Wang R., Win A.N., Li J.N., Chai Y.R. (2018). Genome-wide survey and characterization of fatty acid desaturase gene family in *Brassica napus* and its parental species. Appl. Biochem. Biotechnol..

[64] Lakhssassi N., Zhou Z., Liu S., Piya S., Cullen M.A., El Baze A., Knizia D., Patil G.B., Badad O., Embaby M.G. (2020). Soybean TILLING-by-Sequencing+ reveals the role of novel GmSACPD members in unsaturated fatty acid biosynthesis while maintaining healthy nodules. J. Exp. Bot..

[65] Chu X., Yuan B., Li C., Qian X., Yao Y., Qiao J., Guo D., Wang Z. (2025). Genome-wide analysis of the FAD gene family in *Solanum tuberosum* L. reveals its involvement in cold stress tolerance. Front. Plant Sci..

[66] Chi X., Yang Q., Lu Y., Wang J., Zhang Q., Pan L., Chen M., He Y., Yu S. (2011). Genome-wide analysis of fatty acid desaturases in soybean (*Glycine max*). Plant Mol. Biol. Rep..

[67] Sun J., Chen M., Zhu M., Jiang Y., Meng J., Zhao D., Tao J. (2018). Cloning, characterization, and expression analysis of three FAD8 genes encoding a fatty acid desaturase from seeds of *Paeonia ostii*. Molecules.

[68] Yaeno T., Matsuda O., Iba K. (2004). Role of chloroplast trienoic fatty acids in plant disease defense responses. Plant J..

[69] Yang D., Wang R., Lai H., He Y., Chen Y., Xun C., Zhang Y., He Z. (2024). Comparative transcriptomic and lipidomic analysis of fatty acid accumulation in three *Camellia oleifera* varieties during seed maturing. J. Agric. Food Chem..

[70] Cerone M., Smith T.K. (2022). Desaturases: Structural and mechanistic insights into the biosynthesis of unsaturated fatty acids. IUBMB Life.

[71] Fu C., Fu Q., Wang S., Wu F., Jiang N., Zhou R., Yang Y., Xue Y. (2024). Genome-wide analysis of fatty acid desaturase genes in moso bamboo (*Phyllostachys edulis*) reveal their important roles in abiotic stresses responses. BMC Genom..

[72] Noor J., Ahmad I., Ullah A., Iqbal B., Anwar S., Jalal A., Okla M.K., Alaraidh I.A., Abdelgawad H., Fahad S. (2024). Enhancing saline stress tolerance in soybean seedlings through optimal NH_4_^+^/NO_3_^−^ ratios: A coordinated regulation of ions, hormones, and antioxidant potential. BMC Plant Biol..

[73] Jahan M.S., Zhao C.J., Shi L.B., Liang X.R., Jabborova D., Nasar J., Zhou X.B. (2023). Physiological mechanism of melatonin attenuating to osmotic stress tolerance in soybean seedlings. Front. Plant Sci..

[74] Yuan S., Wu X., Liu Z., Luo H. (2012). Abiotic stresses and phytohormones regulate expression of *FAD2* gene in *Arabidopsis thaliana*. J. Integr. Agric..

[75] Flors V., Ton J., Jakab G., Mauch-Mani B. (2005). Abscisic acid and callose: Team players in defence against pathogens?. J. Phytopathol..

[76] He M., Ding N.-Z. (2020). Plant Unsaturated fatty acids: Multiple roles in stress response. Front. Plant Sci..

[77] Kazaz S., Miray R., Lepiniec L., Baud S. (2022). Plant monounsaturated fatty acids: Diversity, biosynthesis, functions and uses. Prog. Lipid Res..

[78] Guo Q., Liu L., Barkla B.J. (2019). Membrane lipid remodeling in response to salinity. Int. J. Mol. Sci..

[79] Perfileva A.I., Krutovsky K.V. (2024). Effect of manganese- and selenium-containing nanocomposites on soybean resistance to *Pectobacterium carotovorum* and microbial landscape of soybean seedlings. Plant Growth Regul..

[80] Geng L., Li L., Sun X., Cheng S., He J. (2025). Recent advances towards selenium nanoparticles: Synthetic methods, functional mechanisms, and biological applications. Foods.

[81] Zelentsov S.V., Moshnenko E.V., Trunova M.V., Bubnova L.A., Budnikov E.N., Lukomets A.V., Savichenko V.G., Dorofeev N.V., Katysheva N.B., Pomortsev A.V. (2021). A cold-resistant soybean cultivar of the northern ecotype Sayana. Oil Crops.

[82] Perfileva A.I., Zakharova O.V., Graskova I.A., Krutovsky K.V. (2024). Effect of selenium, copper and manganese nanocomposites in arabinogalactan matrix on potato colonization by phytopathogens *Clavibacter sepedonicus* and *Pectobacterium carotovorum*. Plants.

[83] Perfileva A.I., Tsivileva O.M., Nozhkina O.A., Karepova M.S., Graskova I.A., Ganenko T.V., Sukhov B.G., Krutovsky K.V. (2021). Effect of natural polysaccharide matrix-based selenium nanocomposites on *Phytophthora cactorum* and rhizospheric microorganisms. Nanomaterials.

[84] Strekalovskaya E.I., Perfileva A.I., Vyatchina O.F., Stom D.I., Romashchenko A.V., Kasatova A.I., Kon’kova T.V., Sukhov B.G., Krutovsky K.V. (2025). Effect of selenium–arabinogalactan nanocomposite on environmental bacteria. J. Compos. Sci..

[85] Perfileva A.I., Graskova I.A., Sukhov B.G., Krutovsky K.V. (2022). Effect of selenium nanocomposites based on natural polymer matrices on the biomass and storage of potato tubers in a field experiment. Agronomy.

[86] Perfileva A.I., Nozhkina O.A., Graskova I.A., Zabanova N.S., Sukhov B.G. (2021). Phytotoxicity of selenium nanocomposites in natural matrices on potato plant development *in vitro*. Agrokhimiya.

[87] Papkina A.V., Perfileva A.I., Zhivet’yev M.A., Borovskii G.B., Graskova I.A., Klimenkov I.V., Lesnichaya M.V., Sukhov B.G., Trofimov B.A. (2015). Complex effects of selenium-arabinogalactan nanocomposite on both phytopathogen *Clavibacter michiganensis* subsp. *sepedonicus* and potato plants. Nanotechnol. Russ..

[88] Folch J., Lees M., Sloane Stanley G.H. (1957). A Simple method for the isolation and purification of total lipids from animal tissues. J. Biol. Chem..

[89] Nokhsorov V.V., Dudareva L.V., Petrov K.A. (2020). Seasonal dynamics of lipids and their fatty acids in leaf buds of *Betula pendula* Roth and *Alnus alnobetula* subsp. *fruticosa* (Rupr.) Raus under conditions of the cryolithozone. Russ. J. Plant Physiol..

[90] Naraikina N.V., Pchelkin V.P., Tsydendambaev V.D., Trunova T.I. (2020). Changes in fatty acid composition and lipid content occurring in potato leaves during cold hardening: The role of ∆12-acyl-lipid desaturase. Russ. J. Plant Physiol..

[91] Tarasenko T.A., Elizova K.D., Tarasenko V.I., Koulintchenko M.V., Konstantinov Y.M. (2025). Tric proteins and TOM complex subunits are involved in the import of short DNA fragments into *Arabidopsis* mitochondria. Protoplasma.

[92] Mena E., Reboledo G., Stewart S., Montesano M., Ponce de Leon I. (2023). Comparative analysis of soybean transcriptional profiles reveals defense mechanisms involved in resistance against *Diaporthe caulivora*. Sci. Rep..

[93] Lebedev V.G., Korobova A.V., Shendel G.V., Shestibratov K.A. (2023). Hormonal status of transgenic birch with a pine glutamine synthetase gene during rooting *in vitro* and budburst outdoors. Biomolecules.

[94] Veselov S.U., Kudoyarova G.R., Egutkin N.L., Gyuli-Zade V.G., Mustafina A.R., Kof E.K. (1992). Modified solvent partitioning scheme providing increased specificity and rapidity of immunoassay for indole 3-acetic acid. Physiol. Plant..

