# Supplementary material for: The Effect of Selenium–Arabinogalactan Nanocomposite on Fatty Acid Composition in Soybean Seedlings Grown from Pectobacterium carotovorum–Infected Seeds
Source: Plants (Basel). 2026 May 27;15(11):1647. doi: 10.3390/plants15111647 (PMC13259082; doi:10.3390/plants15111647)
Supplement: Supplementary file 1 [file plants-15-01647-s001.zip › plants-4243045-supplementary.pdf]

Table S1. The influence of Se/AG NC on soybean seed FA content. Median [Q1;Q3]

Fatty acid name	Cn:m formula	Control	Se/AG NC
Myristic	C14:0	0.11 [0.09; 0.15]	0.14 [0.11; 0.15]
Pentadecanoic	C15:0	0.04 [0.03; 0.04]	0.03 [0.03; 0.05]
Palmitic	C16:0	19.14 [17.58; 20.37]	18.91 [18.10; 20.16]
Palmetoleic	C16:1 (n-9)	0.13 [0.10; 0.21]	0.12 [0.10; 0.14]
Margaric	C17:0	0.20 [0.15; 0.21]	0.17 [0.14; 0.19]
Stearic	C18:0	4.98 [4.41; 5.17]	4.50 [3.96; 5.52]
Oleic	C18:1 (n-9)	12.60 [10.60; 15.32]	10.68 [5.74; 15.63]
Cis-vaccenic	C18:1 (n-7)	-	4.18 [3.90; 4.47]
Linoleic	C18:2 (n-6)	50.38 [48.57; 52.63]	50.92 [47.90; 54.36]
Linolenic	C18:3 (n-3)	11.43 [11.05; 12.04]	10.86 [10.51; 12.05]
Arachidic	C20:0	0.38 [0.30; 0.43]	0.37 [0.21; 0.61]
Gadoleic (eicosenoic)	C20:1(n-9)	0.09 [0.06; 0.12]	0.05 [0.04; 0.09]
Behenic	C22:0	0.26 [0.10; 0.42]	0.40 [0.21; 0.70]
ΣUSFA		75.06 [74.09; 76.05]	75.15 [74.44; 75.65]
ΣSFA		24.88 [23.85; 25.82]	24.87 [24.28; 25.51]
